# Trimethylamine N-Oxide Derived from a High-Protein Diet Induces Insulin Resistance in Pregnant Mice via Gut Microbiota Remodeling

**DOI:** 10.3390/microorganisms14061356

**Published:** 2026-06-17

**Authors:** Xiaoqian Chen, Kehao Ma, Yichen Shi, Yuhui Li, Yanli Ji, Yehao Liu

**Affiliations:** 1 Department of Health Inspection and Quarantine, School of Public Health, Anhui Medical University, Hefei 230032, China; 2 School of Life and Health Science, Environmental Engineering, Hefei University, Hefei 230601, China

**Keywords:** high protein diet, insulin resistance, trimethylamine, gut microbiota, *Coprococcus*

## Abstract

Insulin resistance (IR) is a significant risk factor for various diseases, particularly during pregnancy. Dietary patterns have been reported to influence IR susceptibility. High-protein (HP) diet has gained popularity for its role in weight management. However, whether trimethylamine N-oxide (TMAO), which is produced in the liver from gut microbiota-derived metabolites of dietary protein, influences IR remains uncertain. In this study, we established a pregnant mouse model to examine the effect of an HP diet on IR, assess its impact on liver function, and investigate associated signaling pathways. The role of gut microbiota was also evaluated. We found that the HP diet induced liver injury in pregnant mice following significantly decreased body weight. The HP diet also elevated plasma TMAO levels and upregulated hepatic *FMO_3_* expression. Transcriptomic analysis revealed enrichment of insulin-related signaling pathways in the HP group, with notable downregulation of the *Insrr* gene. IR was induced through the IRS-1/PI3K/Akt signal pathway. Gut microbiota composition was disrupted in HP group, characterized by an increased Firmicutes/Bacteroidetes ratio and a higher abundance of the TMA-producing genus *Coprococcus*, indicating an elevated potential for TMA generation. Furthermore, several amino acid metabolism pathways closely linked to IR were also enriched in the HP group. In conclusion, our study demonstrates that HP diet induces liver injury and increases IR risk during pregnancy. Gut microbiota contributes to this process, in part through an enhanced capacity for TMA production. These findings highlight the need for greater attention to dietary patterns in pregnancy to mitigate metabolic risks.

## 1. Introduction

With the rising prevalence of obesity, a variety of effective and affordable interventions, including specific dietary patterns alongside physical activity [1], have been implemented. In recent years, dietary patterns have gained increasing attention, with the Mediterranean diet being extensively studied [2]. Meanwhile, the high-protein (HP) diet, characterized by high protein and low carbohydrate intake, has also drawn interest due to its efficacy in weight loss [3]. However, this dietary regimen is generally recommended for non-pregnant populations, and its potential risks for pregnant women remain unclear. Pregnancy is a unique physiological state accompanied by profound metabolic changes, which makes pregnant individuals more vulnerable to dietary disturbances.

Many high protein foods, such as meat and fish, are rich in L-carnitine, choline, and betaine. These compounds are converted by gut microbes into trimethylamine (TMA) [4], which is subsequently oxidized in the liver by hepatic flavin-containing monooxygenase 3 (FMO_3_) to form trimethylamine N-oxide (TMAO) [5]. Elevated TMAO levels have been associated with several chronic diseases, including atherosclerosis, cardiovascular disorders, and neurological conditions [6,7]. Recent evidence also suggests a link between high plasma TMAO and insulin resistance (IR) [8]. Given that IR is a known risk factor for gestational diabetes mellitus (GDM), hypertension, and obesity in pregnant women [9,10,11] and is ultimately associated with adverse pregnancy outcomes [12], it is important to clarify whether HP diet can induce IR during pregnancy.

Gut microbiota plays a crucial role in host metabolism. In turn, dietary patterns can modulate the composition of gut microbiota, thereby influencing host health. For example, a high-fat diet has been shown to increase the Firmicutes/Bacteroidetes ratio, contributing to a higher risk of obesity [13]. Because dietary protein can be converted to TMA in the gut, microbial taxa capable of protein degradation may become enriched. Identifying TMA-producing taxa could deepen our understanding of the role of gut microbiota in metabolic health.

Testosterone levels remain elevated during pregnancy [14]. FMO_3_ is the key hepatic enzyme responsible for converting TMA into TMAO, and its expression is sex-hormone-dependent; androgens such as testosterone upregulate hepatic FMO_3_ expression [15,16]. This hormonal feature distinguishes pregnant subjects from non-pregnant adults and may further amplify TMAO synthesis upon HP diet intervention. Thus, pregnant women may be more susceptible to TMAO-related impacts than non-pregnant women. To evaluate the risks and underlying mechanisms associated with HP diet during pregnancy, we aimed to: (1) determine whether an HP diet induces IR in pregnant mice and elucidate the involved signaling pathways; (2) investigate the role of gut microbiota in the link between HP diet and IR; and (3) identify keystone bacterial taxa in the gut microbiota that may serve as biomarkers for HP diet-induced IR. Understanding the relationship between HP diet and IR risk mediated by gut microbiota could provide new targets for improving metabolic health during pregnancy.

## 2. Materials and Methods

### 2.1. Reagents

All commercial kits used in this study were purchased from Nanjing Jiancheng Biotechnology Co., Ltd. (Nanjing, China). Test strip for blood glucose was purchased from Roche Diagnostics Company (Mannheim, Germany). Antibiotics were purchased from Shanghai Sangon Biotechnology Co., Ltd. (Shanghai, China).

### 2.2. Animals and Experimental Design

All mice were purchased from Beijing Vital River Laboratories (Beijing, China). Animal experiment was approved by the Animal Ethic Committee of Anhui Medical University. All mice were raised in SPF room and given free food and water. After one-week acclimation, all mice were randomly divided into the following two groups (n = 8–10 per group): control group received normal chow (AIN93G, 20% energy from protein); HP group received HP diet (Modified AIN93G with 50% energy from protein). Both normal chow and HP diet were purchased from Xietong Bioengineering Co., Ltd. (Nanjing, China). To investigate the role of gut microbiota in TMAO production and IR, an additional group named AB-treated group, which received HP diet and antibiotic cocktail in drinking water (ampicillin (1 g/L), vancomycin (0.5 g/L) and metronidazole (1 g/L)), was used. Antibiotic cocktail was used to remove gut microbiota. Following a 2-week dietary intervention, female mice were paired with a fertile male mouse for mating. The presence of a copulatory plug was defined as gestational day 1 (GD1), after which male mice were removed, and pregnant females were housed individually throughout the experiment. All pregnant mice were maintained on their respective assigned diets continuously. All functional assessments including OGTT, all sample collections (feces, blood, and liver tissues) for TMA/TMAO detection and hepatic biochemical analysis were performed on gestational day 18 (GD18) during pregnancy.

All pregnant mice were included in the study, with litter size ranging from 6 to 14 pups per litter. Litter size was recorded for each dam, and no significant difference in the number of pups was detected among experimental groups. Litter size was included as a covariate during statistical analysis of maternal body weight.

### 2.3. The Detections of Fecal TMA and Plasma TMAO

Fecal TMA concentration was measured using capillary electrophoresis. Briefly, fecal samples were homogenized in ultrapure water and centrifuged to obtain the supernatant. Approximately 100 µL of supernatant was mixed with 300 µL of acetonitrile containing 80 mM 2,4′-dibromoacetophenone as an internal standard. TMA levels were then quantified using an Agilent 7100 series capillary electrophoresis system (Agilent Technologies, Santa Clara, CA, USA) equipped with a UV–Vis diode array detector.

A commercial ELISA kit, purchased from Shanghai Tongwei Biotechnology Co., Ltd. (Shanghai, China), was used to measure TMAO concentration in blood samples.

### 2.4. Evaluation of Liver Injury

Blood samples were collected and centrifuged to obtain serum. The serum levels of alanine transaminase (ALT), aspartate transaminase (AST), and alkaline phosphatase (ALP) were quantified using commercially available ELISA kits (Nanjing Jiancheng Biotechnology Co., Ltd., Nanjing, China) according to the manufacturer’s instructions.

In parallel, fresh liver samples were fixed in 4% paraformaldehyde, embedded in paraffin, and sectioned at 5 μm thickness. Sections were stained with hematoxylin and eosin (H and E) and examined under a light microscope for histological analysis.

### 2.5. Glucose Tolerance Test and the Measurement of Insulin Concentration

After a 12 h fast, blood samples were collected from all mice, and fasting blood glucose (FBG) was measured using a glucose meter. For the oral glucose tolerance test (OGTT), mice were administered glucose by gastric gavage (0.1 mL/10 g body weight), and blood glucose concentrations were measured at 0, 60, 120, and 180 min.

Blood samples were centrifuged at 4 °C and 400× *g* for 10 min to obtain supernatant. Serum insulin levels were quantified using a commercially available ELISA kit (Nanjing Jiancheng Biotechnology Co., Ltd., Nanjing, China) according to the manufacturer’s instructions.

The homeostatic model assessment for insulin resistance (HOMA-IR) was calculated using the following standard formula: HOMA-IR = FBG (mmol/L) × fasting insulin (mU/L)/22.5.

### 2.6. RNA Extraction and Transcriptome Analysis

Total RNA was extracted from fresh liver samples using TRIzol reagent (Thermo Fisher Scientific, Inc., Waltham, MA, USA) according to manufacturer’s protocol. After removing genomic DNA by DNase I, the quality and quantity of RNA was checked. Qualified RNA samples were sent to Personalbio Technology Co., Ltd. (Shanghai, China) for transcriptome sequencing and bioinformatic analysis.

### 2.7. Quantification of Target Gene Expression by Real-Time PCR

Total RNA was extracted from fresh liver samples using TRIzol reagent (Thermo Fisher Scientific, Inc., Waltham, MA, USA). Complementary DNA (cDNA) was synthesized from 1 µg of total RNA using a PrimeScript RT reagent kit with gDNA Eraser (Takara Biotechnology Co., Ltd., Dalian, China) following the manufacturer’s instructions. Quantitative real-time PCR (qPCR) was performed in a Roche LightCycler 96 thermal cycler (Roche, Basel, Switzerland) with SYBR Green II. The reaction conditions consisted of an initial denaturation at 95 °C for 5 min, followed by 40 cycles of 95 °C for 30 s, 55 °C for 30 s, and 72 °C for 30 s. Primers used in this study are listed in Table S1. β-Actin was used as an internal reference gene, and relative mRNA expression levels were calculated using the 2^−ΔΔCq^ method.

### 2.8. Genomic DNA Isolation and Metagenomic Sequencing Gut Microbiota

Genomic DNA was isolated from stool samples using QIAamp Fast DNA Stool Mini Kit (QIAGEN Company, Hilden, Germany) according to manufacturer’s instruction. After checking the quality of the genomic DNA using Nanodrop 2000 spectrophotometer (Thermo Scientific, Waltham, MA, USA), all qualified DNA samples were used for shotgun metagenomic sequencing. Metagenomic libraries were constructed and sequenced on the Illumina platform by Personalbio Technology Co., Ltd. (Shanghai, China). Raw sequencing data were quality-filtered, assembled, and annotated for species classification and functional analysis using standard metagenomic bioinformatics pipelines.

The depletion efficiency of gut microbiota by antibiotic cocktail was validated via alpha-diversity analysis derived from metagenomic data. Two core indexes, observed species and Shannon index, were calculated to evaluate microbial richness and community diversity across groups. A pronounced drop in both indicators in the AB group was used to confirm effective depletion of intestinal commensal microbes, consistent with previously established validation strategies for antibiotic-treated mouse models.

### 2.9. Statistical Analysis

Data analysis was conducted using Graphpad Prism 9.5. All data are expressed as mean ± standard error of the mean (SEM). Statistical significance was considered at the *p* < 0.05 level.

Two-way analysis of variance (two-way ANOVA) combined with post hoc multiple comparison test was used to analyze serial blood glucose values measured at different time points during OGTT, with group and sampling time as two fixed factors. For single-time-point indicators (serum TMAO, liver enzyme activity, insulin concentration, gene expression, alpha diversity, etc.), one-way ANOVA followed by Tukey’s post hoc test was adopted for intergroup comparisons.

In all bar graphs in this study, different lowercase letters (a, b) above columns represent significant differences between groups (*p* < 0.05), while the same letters indicate no significant difference between the compared groups.

## 3. Results

### 3.1. Effects of HP Diet on Body Weight and Liver Function

**Figure 1:**
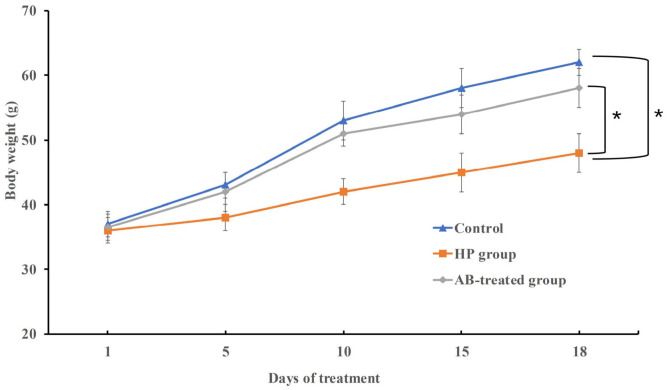
Effect of HP diet on the weight changes in mice; * *p* < 0.05.

An in vivo experiment was conducted to evaluate the effect of a HP diet on body weight in mice. The average litter size was comparable across all groups (*p* > 0.05), indicating that the variation in fetal/pup number did not interfere with the comparison of maternal body weight. After adjusting for litter size, the changes in maternal body weight were calculated. As shown in Figure 1, the final body weight and weight gain of mice in the HP group were significantly lower than those in the control group. Notably, no significant difference in these parameters was observed between the control group and the AB-treated group. These results suggest that gut microbiota is associated with the changes in body weight gain under HP diet intervention.

**Figure 2:**
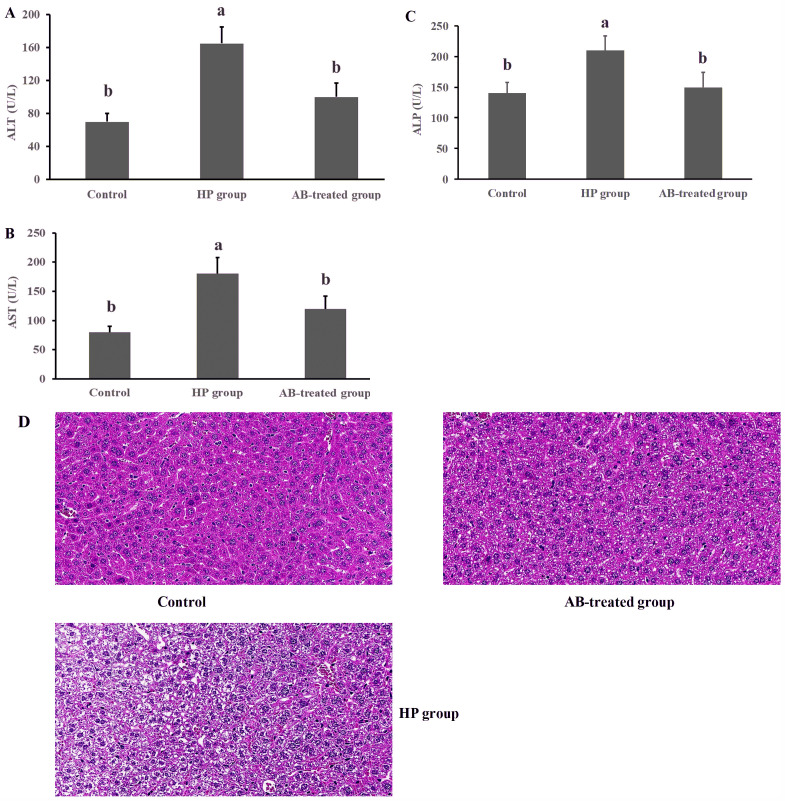
Effects of HP diet on hepatic injury. ALT level (**A**). AST level (**B**). ALP level (**C**). H and E staining of liver sections (**D**). Different lowercase letters indicate significant differences between groups (*p* < 0.05).

Given that TMAO is synthesized in the liver and previous studies have linked elevated TMAO levels to liver injury [8], we evaluated the hepatic effects of the HP diet by assessing liver function and performing histopathological analysis. Measurement of serum biomarkers (ALT, AST, and ALP) revealed a significant increase in ALT activity in the HP group compared with control. Similarly, AST and ALP levels were also markedly elevated in HP mice (Figure 2A–C).

Histopathological analysis of liver tissue was performed to further assess TMAO-associated hepatotoxicity. As shown in Figure 2D, liver sections from the control group exhibited hepatocytes with abundant cytoplasm, large round nuclei, and minimal lipid droplets. The hepatic cord arrangement was compact and orderly, and sinusoids appeared irregular without noticeable dilation; no evident inflammation or structural abnormality was observed. In contrast, liver sections from the HP group displayed widespread hepatocellular edema, with cytoplasm appearing pale and loosely structured. Numerous hepatocytes showed ballooning degeneration, cellular swelling, and cytoplasmic vacuolization. These pathological changes are correlated with hepatic TMAO accumulation, indicating a potential association between HP diet-induced TMAO elevation and hepatic structural damage.

### 3.2. Effects of HP Diet on Blood Glucose and Glucose Tolerance

**Figure 3:**
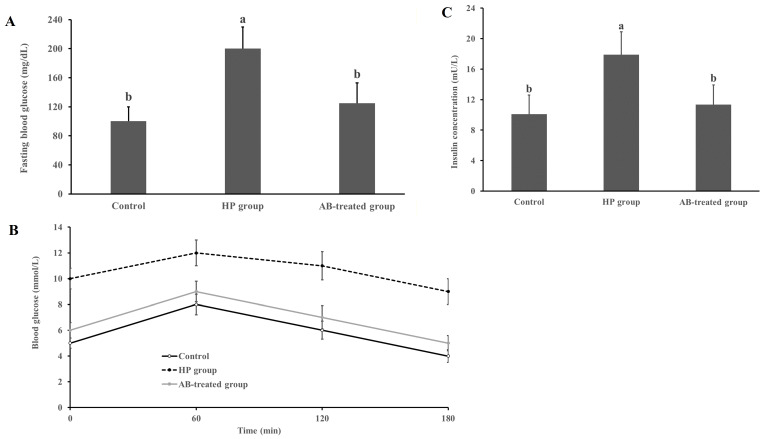
Effect of HP diet on the fasting blood glucose (**A**), OGTT (**B**), and serum insulin (**C**) among the groups. Statistical analysis was performed using two-way ANOVA followed by post hoc multiple comparisons. Different lowercase letters indicate significant differences between groups (*p* < 0.05).

IR is a well-studied risk factor for gestational diabetes mellitus (GDM), and dietary patterns are closely linked to IR development. Abnormalities in fasting blood glucose (FBG) and glucose tolerance serve as key indicators of IR. To evaluate the impact of an HP diet on IR in pregnant mice, we first compared FBG levels among groups. The HP group showed a significant increase in FBG relative to controls (Figure 3A). Further assessment of OGTT revealed that the HP group exhibited a progressive rise in blood glucose after glucose administration, peaking at 1 h and remaining elevated even 2 h post-challenge, Figure 3B indicating marked glucose intolerance. Moreover, fasting serum insulin levels were significantly higher in the HP group, confirming severe IR (Figure 3C). Notably, gut microbiota depletion via antibiotic intervention significantly reversed HP diet-induced increases in FBG and improved glucose intolerance. These phenotypic results indicate that gut microbiota is closely associated with HP diet-induced glucose metabolic disorders and IR in pregnant mice.

### 3.3. Effects of HP Diet on the Potential IR-Related Signaling Pathway

Elevated hepatic level of TMAO is known to exert adverse metabolic effects, including the potential promotion of IR [8]. To identify TMAO-related IR signaling pathways at the transcriptional level, we performed RNA-sequencing analysis of liver tissues. After filtering low-quality reads, an average of 540.06 ± 35.46 million clean reads per sample were obtained. Comparative transcriptomics revealed 750 DEGs, of which 261 were unique to the control group, 151 to the HP group, and 469 were common to both groups.

**Figure 4:**
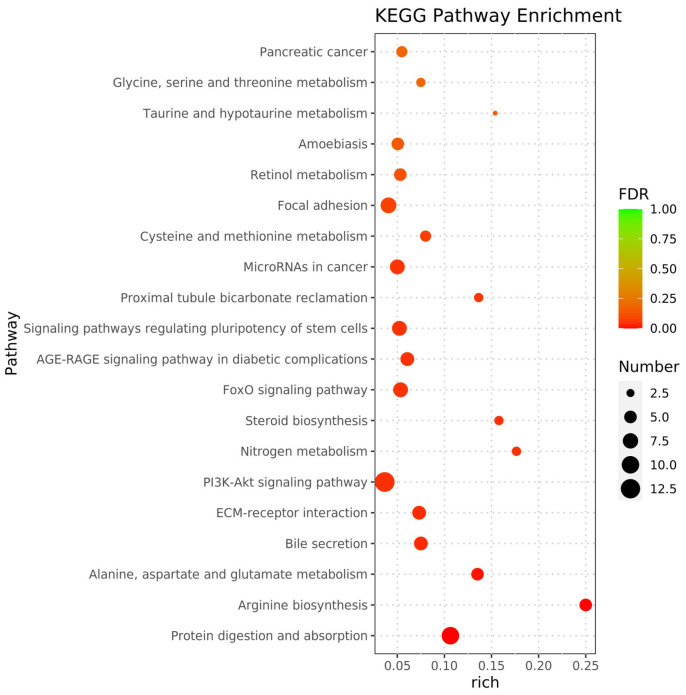
Effect of HP diet on the KEGG pathway enrichment annotations of the DEGs in mice. X-axis indicates statistical significance and Y-axis indicates functional pathways.

To further delineate pathways influenced by the HP diet, we performed KEGG pathway enrichment analysis. A set of 30 pathways was significantly enriched (Figure 4), distributed across the following five major categories: environmental information processing (five pathways), cellular processes (two), human diseases (three), metabolism (four), and organismal systems (five). Notably, the *IRS*-1/*PI3K*/*Akt* signaling pathway, critically involved in insulin signaling, was the enriched terms, suggesting its potential role in HP-diet-associated IR. Subsequent experiments were carried out to validate these findings.

### 3.4. Effects of HP Diet on the IRS-1/PI3K/Akt Signaling Pathway

**Figure 5:**
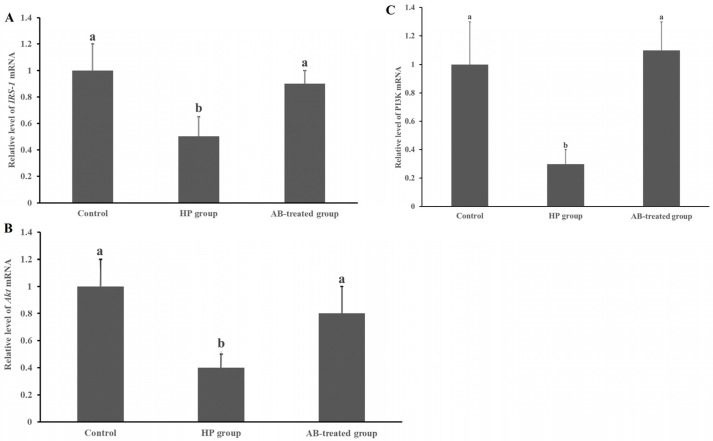
Effect of HP diet on the *IRS-1*/*PI3K*/*Akt* insulin signaling pathway evaluated by mRNA expression level of IRS-1 (**A**), Akt (**B**), and PI3K (**C**). Different lowercase letters indicate significant differences between groups (*p* < 0.05).

As mentioned above, *IRS-1*/*PI3K*/*Akt* signaling pathway was enriched in the KEGG analysis. To elucidate the mechanisms underlying TMAO-related metabolic impacts, the present study investigated key signaling pathways potentially involved using qPCR. As shown in Figure 5, the mRNA expression levels of the key genes in the *IRS*-1/*PI3K*/*Akt* pathway were significantly altered. Compared with the control group, the HP group exhibited marked downregulation in the expression of critical pathway components, including *PI3K*, *Akt*, and *IRS*-1. Interestingly, the expression of these three genes in AB-treated group showed no difference with the control, suggesting the tight relationship of gut microbiota with this signaling pathway.

### 3.5. Effects of HP Diet on TMAO Level in Plasma and FMO_3_ Gene Expression in the Liver

**Figure 6:**
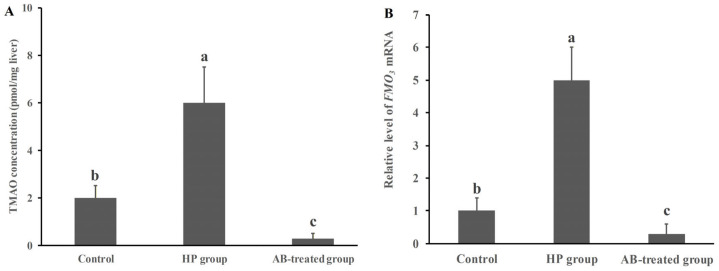
Effect of HP diet on the hepatic TMAO level (**A**) and hepatic *FMO_3_* mRNA expression (**B**) in mice. Different lowercase letters indicate significant differences between groups (*p* < 0.05).

Given that TMAO is a hepatic metabolite of TMA, which is produced from dietary precursors by gut microbiota [17], we hypothesized that HP diet would elevate plasma TMAO level. To test this, we measured plasma TMAO concentration and hepatic expression of *FMO_3_* gene, the enzyme responsible for converting TMA to TMAO. As shown in Figure 6A, the HP group exhibited a significant increase in plasma TMAO compared with controls. Correspondingly, hepatic *FMO_3_* gene expression was also markedly upregulated in HP group (Figure 6B). Interestingly, after wiping out gut microbiota using antibiotic cocktail, the concentration of TMAO was significantly lower, and the expression of *FMO_3_* gene was markedly downregulated in AB-treated group. These results demonstrate that HP diet intervention is associated with elevated hepatic FMO_3_ expression and increased circulating TMAO levels in pregnant mice.

### 3.6. Effects of HP Diet on the Composition of the Gut Microbiota

Given the pivotal role of gut microbiota in TMA production [18], we compared the gut microbial structure between the HP and control groups. Stool samples (n = 6 per group) were subjected to 16S rRNA gene sequencing, yielding 41,519–44,915 reads per sample. Rarefaction curves indicated that sequencing depth was sufficient to assess microbial diversity.

**Figure 7:**
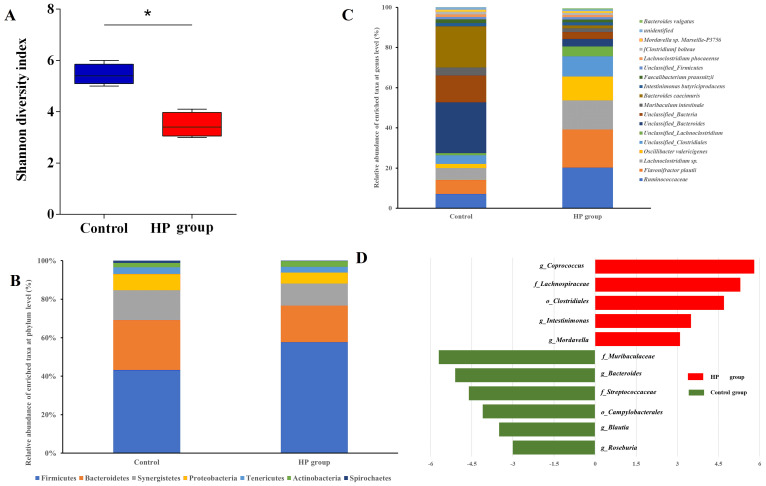
The comparisons of gut microbiota between the two groups. Shannon index (**A**), the relative abundance of predominant taxa at the phylum level (**B**) and genus level (**C**), the most enriched taxa between control and HP group which is determined through LDA score according to linear discriminant analysis effect size (LEfSe) analysis. Green bars indicate that taxa are enriched in the control, and red bars indicate that taxa are enriched in HP fed group (**D**). * *p* < 0.05.

Alpha diversity, evaluated using the Shannon index, was significantly lower in the HP group than in control (Figure 7A), suggesting reduced microbial species richness. Taxonomic profiling revealed that the HP group exhibited a higher relative abundance of Firmicutes at the phylum level (Figure 7B). At the genus level (Figure 7C), unclassified *Ruminococcaceae*, *Flavonifractor*, and *Lachnoclostridium*—all members of the Firmicutes phylum—were notably enriched.

Linear discriminant analysis Effect Size (LEfSe) further identified differentially abundant taxa between the groups (Figure 7D). The HP group showed enrichment of several Firmicutes taxa, including *Coprococcus*, *Lachnospiraceae*, *Clostridiales*, *Intestinimonas*, and *Mordavella*. In contrast, the control group was enriched in *Muribaculaceae* (belonging to Bacteroidetes), *Bacteroides* (belonging to Bacteroidetes), *Streptococcaceae* (belonging to Firmicutes), *Campylobacterales* (belonging to Proteobacteria), *Blautia* (belonging to Firmicutes), and *Roseburia* (belonging to Firmicutes). These results confirm that HP diet significantly reshapes gut microbiota structure and leads to Firmicutes-dominated microbial dysbiosis.

### 3.7. Effects of HP Diet on the Potential of Gut Microbiota to Metabolize Protein

Alterations in gut microbiota composition are known to influence its functional capacity [13]. To characterize functional shifts associated with the HP diet, we annotated microbial genes using the KEGG database (https://www.kegg.jp/kegg/pathway.html, 15 October 2025). All DEGs from both groups mapped to 425 KEGG orthology (KO) entries, with 305 enriched in the HP group and 120 in the control group. We then performed KEGG pathway enrichment analysis focusing on carbon and nitrogen metabolism, as dietary protein serves as a key substrate for microbial nutrient utilization. A total of 115,081 genes were assigned to carbohydrate metabolism and 67,238 to amino acid metabolism.

**Figure 8:**
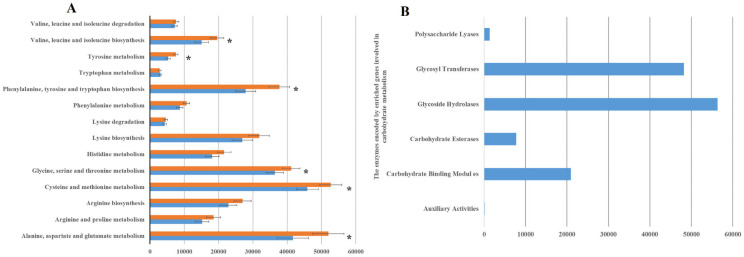
Comparisons of the gene number involved in the metabolism of amino acids (**A**) and carbohydrates (**B**) between the two groups. X-axis indicates gene number and Y-axis indicates the metabolic pathway. * *p* < 0.05.

Specifically, six KEGG pathways related to amino acid metabolism were enriched in the HP group (Figure 8A), including biosynthesis of valine, leucine, and isoleucine, as well as metabolism of tyrosine, phenylalanine, cysteine, methionine, alanine, aspartate, and glutamate. In carbohydrate metabolism, enriched genes primarily encoded glycosyl transferases and glycoside hydrolases (Figure 8B). These results indicate that HP diet intervention is associated with enhanced microbial amino acid and protein metabolic capacity.

### 3.8. Coprococcus Is Positively Correlated with IR

Increased dietary protein elevates intestinal TMA level by providing abundant substrate, thereby raising plasma TMAO concentration. To assess the metabolic impact of TMAO, we evaluated its correlation with insulin levels and HOMA-IR. Significant positive associations were observed between TMAO and both insulin (R = 0.21, *p* = 0.007) and HOMA-IR (R = 0.52, *p* = 0.009), indicating that higher TMAO levels are linked to insulin resistance.

Given the central role of gut microbiota in TMA generation, we performed Spearman correlation analysis to identify taxa associated with HOMA-IR, using all taxa previously enriched in LEfSe analysis. As summarized in Table 1, HOMA-IR showed a positive correlation with the abundance of *Coprococcus*, a known TMA-producing genus. This finding was further supported by qPCR data (Figure S1), which confirmed that *Coprococcus* abundance was significantly higher in the HP group compared with control. Collectively, these correlation results suggest that enriched *Coprococcus* is a potential microbial biomarker associated with HP diet-induced insulin resistance, rather than confirming direct causal effects.

## 4. Discussion

IR is a risk factor for multiple metabolic disorders, including type 2 diabetes, hypertension, and dyslipidemia [9,19,20]. In pregnancy, maternal IR can lead to gestational diabetes mellitus (GDM), increasing the risk of adverse outcomes such as macrosomia, birth injury, and neonatal hypoglycemia [21,22]. In the present study, we demonstrated that high-protein diet induced both IR and liver injury in pregnant mice, whereas control mice did not exhibit these alterations. Notably, with the metabolic disturbances, significant difference in body weight gain was observed between the two groups. While prior studies have suggested that HP diets may improve IR compared with other dietary patterns such as the Mediterranean diet [23], our findings in pregnant mice contrast with those reports. The occurrence of IR in our model appears to be closely linked to pregnancy-associated upregulation of hepatic FMO_3_ expression, a key enzyme that catalyzes the oxidation of TMA to TMAO [5]. These results highlight the need for careful consideration of dietary protein intake during pregnancy to mitigate IR-related complications.

The liver serves as a primary site for glucose metabolism and insulin action [24]. Insulin regulates glucose homeostasis largely through the *IRS-1*/*PI3K*/*AKT* signaling pathway [20]. In pregnant mice fed a HP diet with restricted carbohydrate intake, we observed disruption of this pathway, accompanied by the development of IR. This aligns with previous reports indicating that low-carbohydrate regimens can promote IR and metabolic acidosis even in healthy lean individuals [25]. Impaired insulin-receptor binding and signal transduction are established mechanisms underlying IR [26]. Consistent with this, our transcriptomic data revealed downregulation of the *Insrr* gene, which modulates insulin-receptor interaction [27], and KEGG enrichment analysis confirmed significant alterations in insulin-related signaling pathways. Collectively, these data suggest that HP diet interferes with the *IRS-1*/*PI3K*/*AKT* axis, at least in part by attenuating insulin-receptor binding. Meanwhile, disruption of this pathway, as observed in HP-fed pregnant mice, not only promotes IR but is also closely associated with hepatocellular damage, indicated by elevated serum markers of liver injury (ALT, AST, and ALP) and histopathological alterations including steatosis and ballooning degeneration. This finding aligns with a growing body of evidence highlighting the bidirectional interplay between hepatic injury and IR.

Gut microbiota composition is a critical determinant of TMA production [17]. Our analysis revealed reduced microbial diversity in HP-fed mice, indicating that dietary protein profoundly shapes the gut ecosystem. Similar restructuring has been reported in response to high-fat diets, pharmaceuticals, and other interventions [28]. Shifts in dominant phyla—particularly the Firmicutes/Bacteroidetes ratio—are often associated with energy harvest efficiency, with obesity commonly characterized by an increased Firmicutes/Bacteroidetes ratio [29,30]. Although we observed a higher relative abundance of Firmicutes in HP-fed mice, no corresponding increase in body weight was detected, which may be attributable to the concomitant restriction of dietary carbohydrates limiting fat accumulation. Notably, the HP-fed group displayed significant enrichment of Firmicutes and Actinobacteria—both known to harbor TMA-producing taxa—along with a marked reduction in Bacteroidetes and Euryarchaeota, which generally lack TMA-forming capacity [4,31]. The elevated Firmicutes/Bacteroidetes ratio coincided with increased plasma TMAO levels, supporting the concept that HP-induced microbial remodeling enhances TMA-generating potential [32].

Alterations in microbial community structure are often reflected in functional changes [33]. Metagenomic analysis revealed distinct enrichment of amino acid metabolism pathways in HP-fed mice, consistent with the high protein substrate availability. Several amino acids derived from dietary protein have been implicated in obesity, type 2 diabetes, and immune regulation [34,35,36]. In our study, pathways related to the biosynthesis and metabolism of valine, leucine, isoleucine, tyrosine, phenylalanine, cysteine, methionine, alanine, aspartate, and glutamate were significantly augmented. These microbially produced amino acids can be absorbed by the host and participate in host–microbiota crosstalk. Elevated circulating branched-chain and aromatic amino acids are recognized biomarkers of IR and ectopic lipid accumulation [37,38]. For instance, tyrosine-derived dopamine and L-DOPA can inhibit glucose-stimulated insulin secretion, thereby defending against hypoglycemia [39]; enhanced tyrosine metabolism may thus perturb glucose regulation. The observed enrichment of aromatic amino acid biosynthesis pathways in HP-fed mice further corroborates the link between microbial amino acid metabolism and host IR. Together, these functional insights reinforce the conclusion that HP diet promotes a microbial metabolic landscape conducive to TMA formation and subsequent IR progression.

## 5. Conclusions

This study demonstrates that a high-protein diet-related elevation of circulating TMAO is closely associated with insulin resistance and hepatic morphological and functional abnormalities in pregnant mice, accompanied by altered body weight gain. High-protein diet intervention reshapes the composition and metabolic function of gut microbiota, which correlates with enhanced microbial TMA-producing capacity and subsequent TMAO accumulation. Our findings highlight that gut microbiota dysbiosis mediates the association between high-protein diet and gestational insulin resistance. Furthermore, the increased abundance of *Coprococcus* may serve as a potential microbial biomarker correlated with high-protein diet-related metabolic disorders and IR during pregnancy.

Several limitations of this study should be noted. First, the precise mechanisms through which signal pathway TMAO contributes to IR remain incompletely understood. Second, effects of TMAO beyond IR in pregnant mice have not been comprehensively examined. Third, the conclusions drawn are based solely on animal models and lack supporting evidence from human cohort studies. Fourth, the lack of a normal diet + antibiotic-treated control group limits our ability to fully separate the effects of microbiota depletion from those of the HP intervention. Finally, another limitation is the lack of detection of serum inflammatory markers (IL-6, TNF-α, and CRP). Incorporating these indicators would better elucidate the interplay among gut microbiota, TMAO and insulin resistance. We will include inflammatory index analysis in subsequent experiments to deepen the mechanistic exploration.

## Figures and Tables

**Table 1:** The relationship between enriched taxa and HOMA-IR.

The Abundance of Enriched Taxa	rho	*p* Value
*g_Coprococcus*	0.153	**0.002**
*f_Lachnospiraceae*	0.341	0.061
*o_Clostridiales*	0.236	0.414
*g_Intestinimonas*	0.173	0.128
*g_Mordavella*	0.313	0.273
*f_Muribaculaceae*	0.302	0.307
*g_Bacteroides*	−0.079	0.405
*f_Streptococcaceae*	−0.085	0.517
*o_Campylobacterales*	−0.043	0.617
*g_Blautia*	0.023	0.072
*g_Roseburia*	0.032	0.081

The bolded *p* value in the table is retained to mark the sole statistically significant result (*p* < 0.05) across all data in this table.

## Data Availability

The original contributions presented in this study are included in the article/Supplementary Material. Further inquiries can be directed to the corresponding authors.

## Supplementary Materials

The following supporting information can be downloaded at https://www.mdpi.com/article/10.3390/microorganisms14061356/s1, Figure S1: Relative abundance of the *Coprococcus* genus, calculated as the ratio of *Coprococcus* abundance to the total bacterial abundance, was compared between groups; Figure S2: Effects of Antibiotic Cocktail on Alpha Diversity of gut microbiota. Table S1: The primers used in this study.
