# Supplementary material for: Trimethylamine N-Oxide Derived from a High-Protein Diet Induces Insulin Resistance in Pregnant Mice via Gut Microbiota Remodeling
Source: Microorganisms. 2026 Jun 17;14(6):1356. doi: 10.3390/microorganisms14061356 (PMC13304072; doi:10.3390/microorganisms14061356)
Supplement: Supplementary file 1 [file microorganisms-14-01356-s001.zip › Figure S1.pdf]

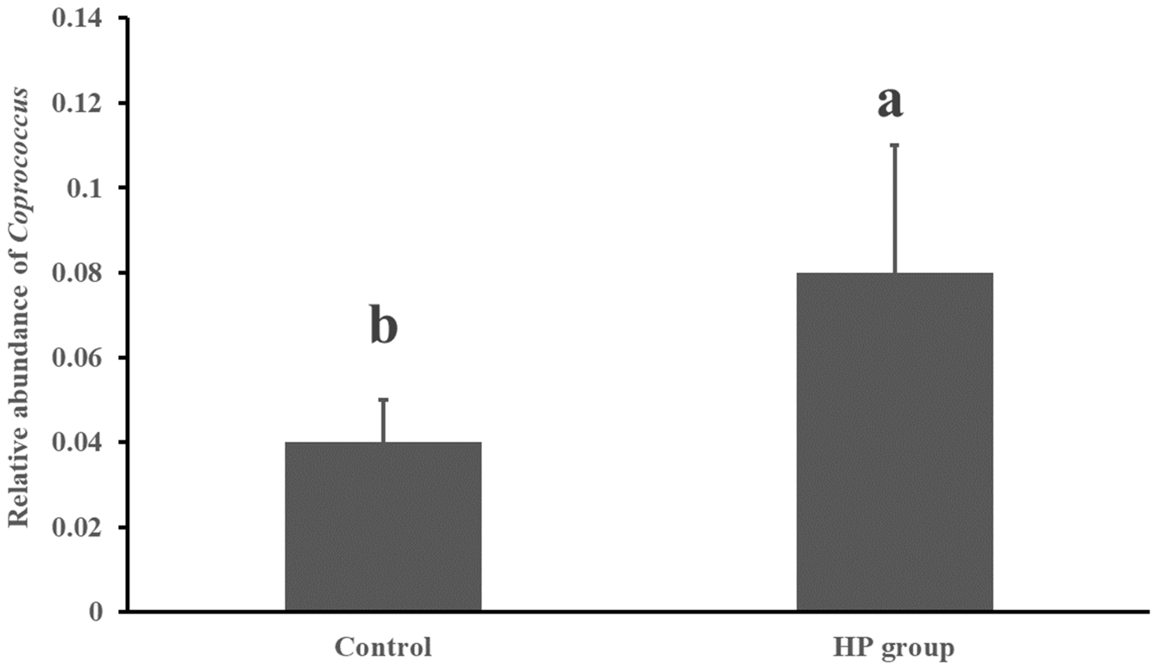

Figure S1. Relative abundance of the *Coprococcus* genus, calculated as the ratio of *Coprococcus* abundance to the total bacterial abundance, was compared between groups. Different letters show significant differences. Significance at $P < 0.05$.
