# Supplementary material for: Trimethylamine N-Oxide Derived from a High-Protein Diet Induces Insulin Resistance in Pregnant Mice via Gut Microbiota Remodeling
Source: Microorganisms. 2026 Jun 17;14(6):1356. doi: 10.3390/microorganisms14061356 (PMC13304072; doi:10.3390/microorganisms14061356)
Supplement: Supplementary file 1 [file microorganisms-14-01356-s001.zip › Figure S2.pdf]

Alpha diversity (observed species and Shannon index) was calculated from metagenomic data to verify gut microbiota depletion after antibiotic intervention.

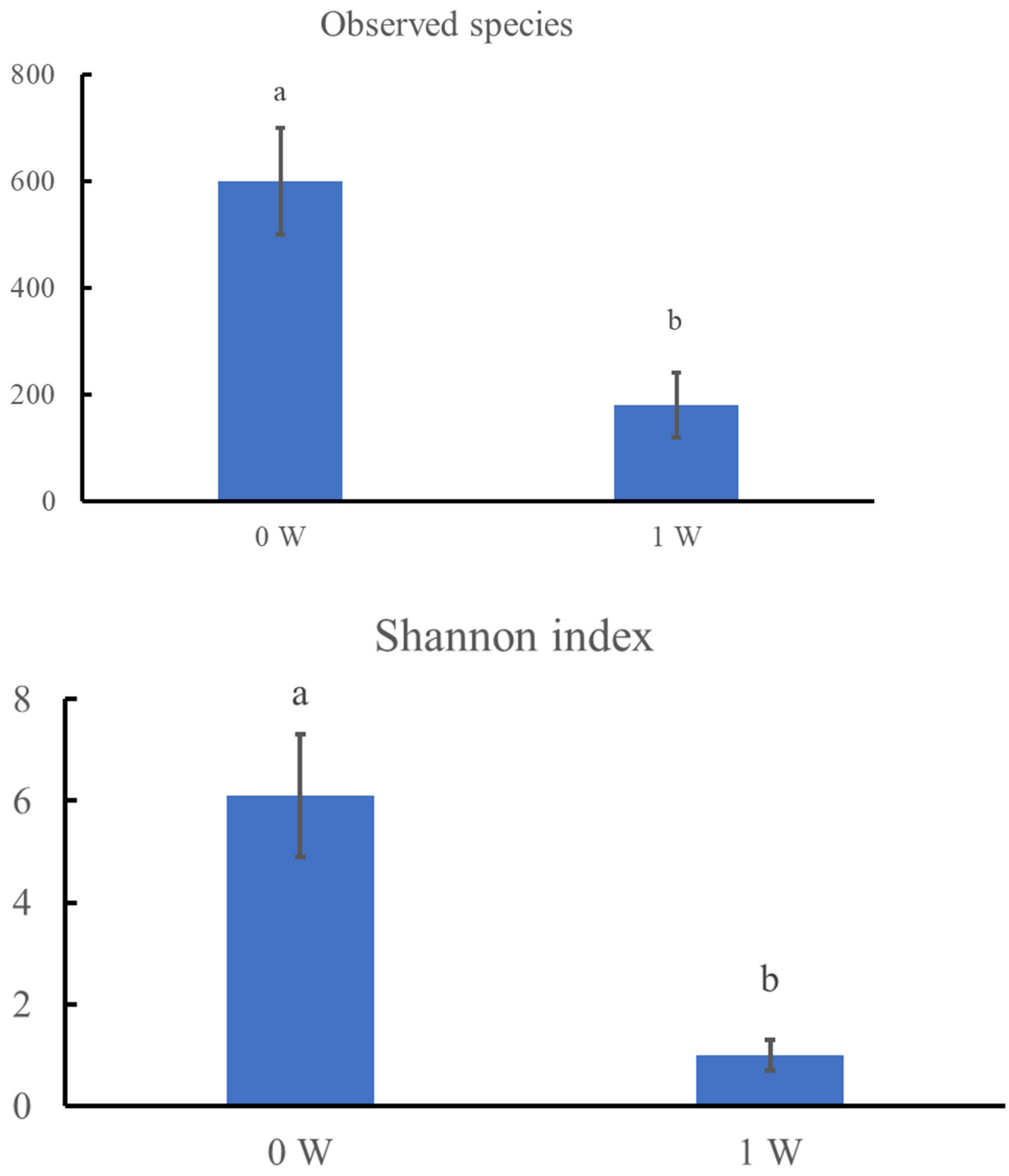

Figure S2. Effects of Antibiotic Cocktail on Alpha Diversity of gut microbiota. Bars with different letters differ significantly ($P < 0.05$). Fecal samples were collected at baseline (0 week, 0 W) and at week 1 (1 W) after the initiation of drug intervention.
