# Supplementary material for: Trimethylamine N-Oxide Derived from a High-Protein Diet Induces Insulin Resistance in Pregnant Mice via Gut Microbiota Remodeling
Source: Microorganisms. 2026 Jun 17;14(6):1356. doi: 10.3390/microorganisms14061356 (PMC13304072; doi:10.3390/microorganisms14061356)
Supplement: Supplementary file 1 [file microorganisms-14-01356-s001.zip › Table S1.pdf]

Table S1. The primers used in this study

Gene name	Primer sequence (5'-3'')
Total bacteria	Forward (F): GCAGGCCTAACACATGCAAGTC<br>Reverse (R): CTGCTGCCTCCCGTAGGAGT
Coprococcus	F: AGCTTGCTCCGGCYGATTTA<br>R: CGGTTTTACCAGTCGTTTCCAA
PI3K	F: ATCGACCTACACTTGGGGGA<br>R: CAATATCTTCTGGCCGGGCT
Akt	F: AGGAGGTCATCGTTGCCAAG<br>R: GGTCGTGGGTCTGGAATGAG
IRS-1	F: TACCGCAACTGCCGAAGATT<br>R: GGGGATGTGTGACGCTAGAC
GAPDH	F: ACAGCAACAGGGTGGTGGAC<br>R: TTTGAGGGTGCAGCGAACTT
